# Hydromorphological and socio-cultural assessment of urban rivers to promote nature-based solutions in Jarabacoa, Dominican Republic

**DOI:** 10.1007/s13280-021-01565-3

**Published:** 2021-06-01

**Authors:** Gonzalo Pradilla, Georg Lamberty, Johannes Hamhaber

**Affiliations:** 1 Institute of Applied Geosciences, Technische Universität Darmstadt, Schnittspahnstrasse 9, 64287 Darmstadt, Germany; 2 Faculty of Spatial Development and Infrastructure Systems, Cologne University of Applied Sciences, Robertstrasse 2, 53111 Cologne, Germany

**Keywords:** Blue and green infrastructure, Latin America and the Caribbean, Nature-based solutions, Rapid stream assessment, River restoration, Sustainable urban planning

## Abstract

In Latin America and the Caribbean, river restoration projects are increasing, but many lack strategic planning and monitoring. We tested the applicability of a rapid visual social–ecological stream assessment method for restoration planning, complemented by a citizen survey on perceptions and uses of blue and green infrastructure. We applied the method at three urban streams in Jarabacoa (Dominican Republic) to identify and prioritize preferred areas for nature-based solutions. The method provides spatially explicit information for strategic river restoration planning, and its efficiency makes it suitable for use in data-poor contexts. It identifies well-preserved, moderately altered, and critically impaired areas regarding their hydromorphological and socio-cultural conditions, as well as demands on green and blue infrastructure. The transferability of the method can be improved by defining reference states for assessing the hydromorphology of tropical rivers, refining socio-cultural parameters to better address river services and widespread urban challenges, and balancing trade-offs between ecological and social restoration goals.

**Supplementary Information:**

The online version contains supplementary material available at 10.1007/s13280-021-01565-3.

## Introduction

The conventional way to manage water in urban systems, based on hard engineering measures while sealing surfaces in city areas, has led to urban problems and water imbalances (Eckart et al. 2017). The related negative consequences, termed urban stream syndrome, include sudden rise and fall of the hydrograph, elevated concentrations of nutrients and contaminants, altered channel morphology and stability, and reduced biotic richness, often accompanied by other symptoms, such as reduced baseflow or a decreased net ecosystem metabolism (Walsh et al. 2005; Wantzen et al. 2019).

As a result, urban water management is shifting from gray-engineered infrastructures towards a new approach that combines the latter with ecosystem management strategies that enhance the blue-green infrastructure (BGI) of cities in an integrative manner (Kabisch et al. 2016). BGI provides multiple ecosystem services for citizens by integrating natural and semi-natural systems such as forests, rivers, floodplains, wetlands, parks, gardens, greenways, and green roofs, inside or connected to a city, with gray infrastructure (Haase 2015; Voskamp and Van de Ven 2015).

Simultaneously, the social and ecological benefits of rivers are increasingly acknowledged and different measures to restore their ecological integrity have been fostered (Kaushal et al. 2015; Pan et al. 2016). Besides the ecological aspects, river restoration addresses multiple co-benefits such as public health, economic value, quality of life, regional regeneration, and disaster risk reduction (Everard and Moggridge 2012). In that sense, BGI and restoration of urban rivers contribute to the overarching framework of nature-based solutions (NbS). This framework comprises actions and projects that aim to protect, restore, create, design, manage, and use ecosystems and semi-natural elements to address societal challenges in a sustainable manner through their multifunctionality (Cohen-Shacham et al. 2016; Kabisch et al. 2016; Faivre et al. 2017).

Citizens build their sense of community, identity, place, and well-being in relation to rivers, green spaces, and other natural elements (Völker and Kistemann 2013). However, a project deemed ecologically successful might not always be desirable for local communities. Therefore, planners and decision-makers must be aware of the tensions and trade-offs between ecological, social, aesthetic, and urban restoration goals (Cockerill and Anderson 2014). With urban communities being highly heterogeneous, their perceptions and uses of urban spaces depend on socio-cultural contexts, e.g., elderly people appreciate the health benefits of urban green spaces, while insecurity for women determines their access to public places (Paul and Nagendra 2017). Thus, participation and trust constitute key social factors that can greatly influence the process, outcomes, and success of river restoration projects (Metcalf et al. 2015).

Methodologically, conventional ecological river assessments either rely on bioindicator species used as proxies for habitat quality or on comprehensive indexes which calculate a cumulative score based on multiple criteria, typically related to hydrological and geomorphological characteristics, riverbank conditions, water quality, and land/aquatic biota. One widely used approach is to assess the hydromorphological and ecological status of water bodies through the deviation from a reference state, also regarded as near-natural or least disturbed condition, selected based on knowledge of the changes caused by anthropogenic activities (Feio et al. 2014). Since this approach is solely focused on the biophysical characteristics of water bodies, different authors and institutions such as the European Commission have advocated for complementing the multi-criteria indexes to incorporate characteristics of the built environment and the effects of socioeconomic conditions on rivers (Davenport et al. 2004; König 2011; Li et al. 2013; Zhao et al. 2019). So far, most of these methodologies are designed for temperate climates and applied in countries of high development status.

In the Global South, river restoration initiatives appear to be increasing, but not systematically documented or analyzed (Bozelli 2019). Thus, the applicability of restoration concepts and related assessment methodologies is still not well understood (Ramírez et al. 2009; Capps et al. 2016; Wantzen et al. 2019).

Currently, there is a plan to revitalize areas at the Yaque del Norte River Basin and implement urban green and blue infrastructure projects in Santiago de Los Caballeros (Santiago City Council 2019), the first of its kind in the Dominican Republic. In the town of Jarabacoa, since 2013 efforts to restore the river and some of its tributary streams are being carried out jointly by the local river basin organization Plan Yaque, The Nature Conservancy, and the Charles River Watershed Association.

The objective of this study is to introduce a combined ecological and social river assessment approach specifically designed towards urban river management in the Global South by employing it in a select case study and assessing its applicability. The method combines the evaluation of hydromorphological and socio-cultural conditions of three streams in and near the town of Jarabacoa, Dominican Republic. To that end, a rapid hydromorphological assessment method widely used in Germany (LAWA-OS) is transferred to the context of a tropical country, complemented by the assessment of selected socio-cultural river features. These assessments, combined with a citizen survey on BGI use and perception, provide initial low-cost and spatially explicit information needed by decision-makers and local stakeholders to identify potential conservation areas along urban river corridors. The applicability and limitations of these methods regarding river restoration efforts in tropical Latin America are discussed. Additionally, an example of how these assessments can be used to prioritize sites and measures is presented.

## Materials and methods

To identify restoration sites and measures of high priority, the current hydromorphological and socio-cultural status was recorded using two visual rapid assessment methods. These methods provided indications of hydromorphological and socio-cultural deficits and potentials along the streams based on a five-level qualitative scale. The assessment results served as input variables for prioritizing river segments based on nine criteria for suitability regarding river restoration. To relate this prioritization to the needs of the population, a survey with 36 questions on demographic indicators, and the citizens’ perception of blue and green infrastructure was conducted.

### Research area

**Fig. 1:**
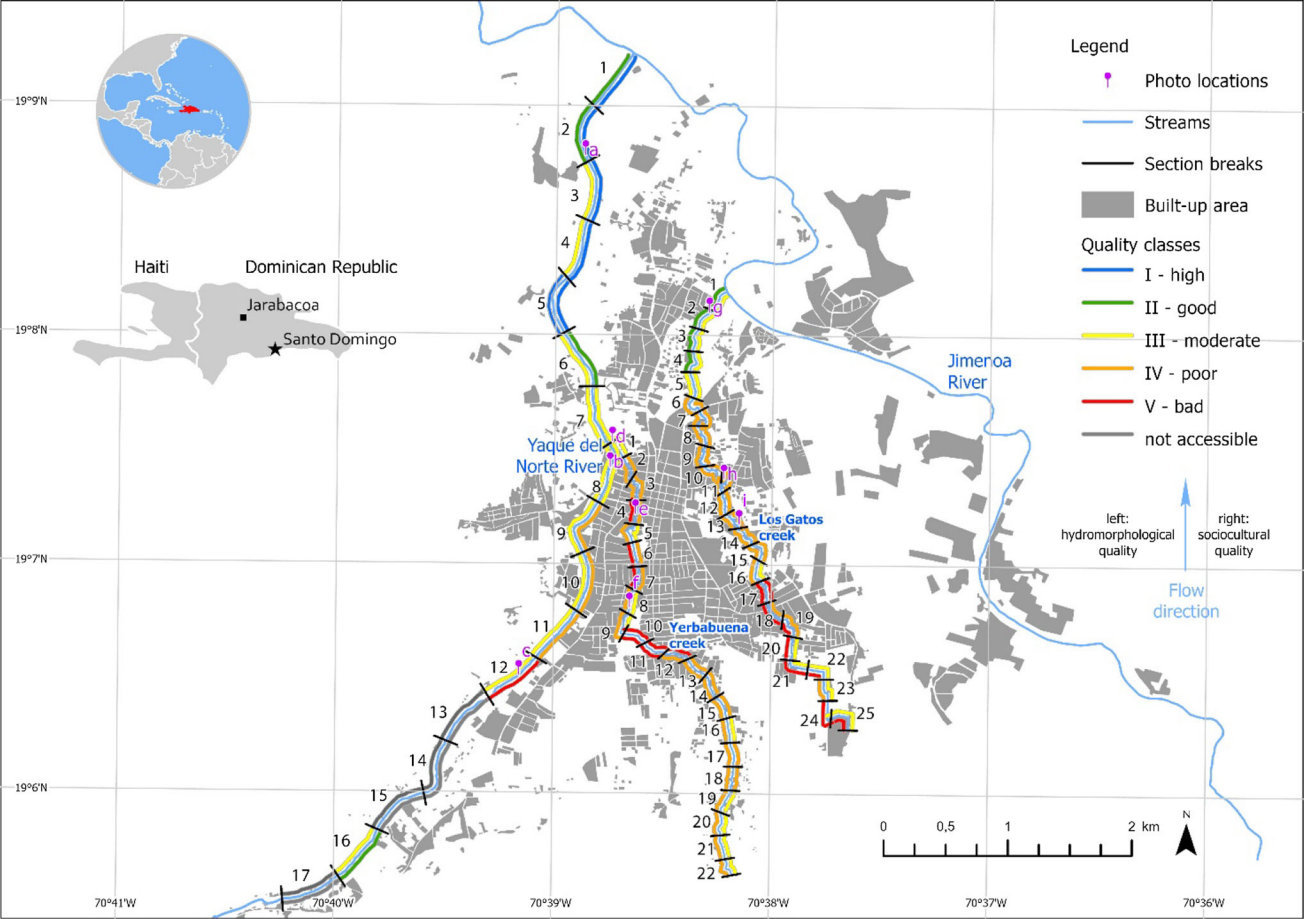
The figure shows the results of the hydromorphological and socio-cultural quality assessment of the three selected streams in Jarabacoa through a 5-class color scheme (numbers indicate the stream sections of 500 m length for Yaque del Norte River and 200 m length for Yerbabuena creek and Los Gatos creek, and letters indicate the position of photos shown in Fig. 2)

The study was conducted at the Yaque del Norte River (hereafter Yaque River) and two of its tributaries in the town of Jarabacoa (56 800 inhabitants), located in the Central Region of the Dominican Republic (19° 07′ 12′′ N and 70° 38′ 24′′ O) (Fig. 1). The town is situated at 527 m a. s. l., the average annual temperature and precipitation are 22.0 °C and 1466.1 mm, respectively (SGN 2010).

**Fig. 2:**
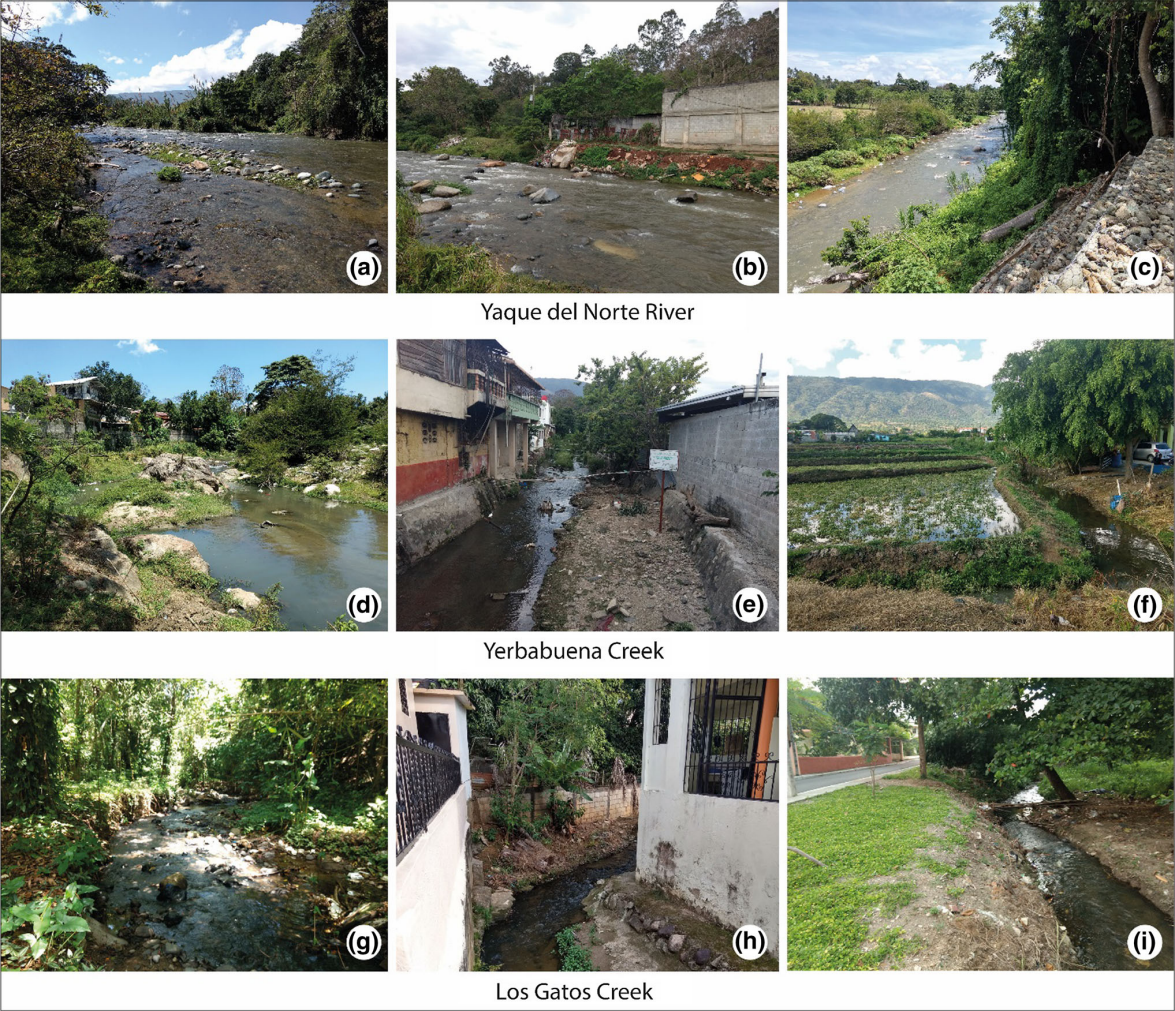
Heterogeneous hydromorphological and socio-cultural quality of the three assessed streams (letters indicate the position within the research area; see Fig. 1)

The area is well known for its mild climate, mountainous landscape, tropical forests, and plentiful rivers and waterfalls, leading Jarabacoa to be known as the ‘green city’ of the Dominican Republic (Corral 2011). The town has developed in the valley and depression between Yaque River and one of its affluents, the Jimenoa River. Three additional watercourses flow through the town: Baiguate River, Los Gatos creek (both affluents of the Jimenoa River), and Yerbabuena creek (affluent of the Yaque River). Here, only those subject to monitoring and restoration plans were included in this study: Yaque River, Yerbabuena creek, and Los Gatos creek (Fig. 2).

The Yaque del Norte is the largest river basin of the Dominican Republic, with a total extension of approximately 7000 km^2^ and a total length of 296 km. The 5.6-km-long Yerbabuena creek originates in the hills south of Jarabacoa, mouthing in the Yaque River close to the town center of Jarabacoa. The source of Los Gatos creek is not clearly known, as the original upstream course has been heavily modified in the past. After 5 km of flowing entirely through the urban area the creek mouths into the Jimenoa River.

### Hydromorphological and socio-cultural assessment

The hydromorphological assessment is largely based on the German LAWA-OS protocol (LAWA 2000; Meier et al. 2013). The method divides streams into sections of 100 m, 200 m, 500 m, or 1000 m length depending on the stream width. The hydromorphological quality of each section is assessed via 7 main parameters composed of a total of 25 single parameters (Table 1 and Fig. S1).

**Table 1:** Main parameters of the two river assessment methods

Hydromorphological stream quality (main and single parameters)	Socio-cultural stream quality
**1. Channel development** 1.1 Curvature 1.2. Erosion at bends 1.3. Longitudinal bars 1.4. Special channel structures **2. Longitudinal profile** 2.1 Transverse constructions 2.2. Backwater 2.3 Piping 2.4 Transverse bars 2.5 Flow diversity 2.6 Depth variation **3. Cross profile** 3.1 Profile type 3.2 Profile depth 3.3 Width erosion 3.4 Width variation 3.5 Culvert/bridge **4. River bed structure** 4.1 Bed substrate 4.2 Bed protection 4.3 Substrate diversity 4.4 Special riverbed structures **5. River bank structure** 5.1 River bank vegetation 5.2 River bank protection 5.3 Special river bank structures **6. Adjacent land zone** 6.1 Land use 6.2 Riparian buffer strip 6.3 Harmful land structures	**Integration in urban setting** **1. Visibility:** Distance from which the river is visible. **2. Reachability:** Paths, roads, and public transport leading to the river. **3. Accessibility**: Degree to which people can directly interact with the river corridor and the water. **Attractiveness of riverine area**: **4. Peculiarity:** **Aesthetics and recognition value of the river corridor.** – Positive factors (historical buildings, floral/faunistic peculiarities, backdrop effect) – Negative factors (degraded natural features, dull infrastructure, monotonous view) **5. Amenity:** Degree to which being at and enjoying the river is possible and comfortable. – Positive factors (e.g., recreation, meeting point, activities, seating, and shaded areas) – Negative factors (e.g., traffic noise, bad smell, trash, bad water quality)

**Fig. 3:**
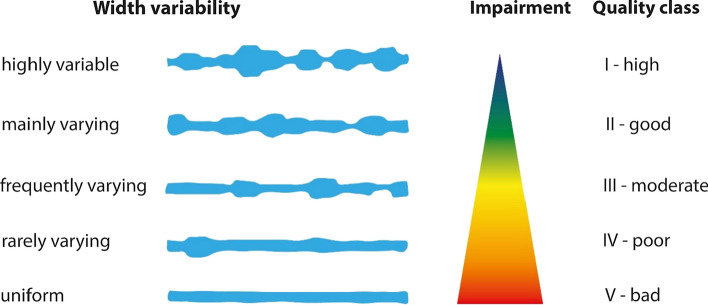
Example of hydromorphological assessment: The parameter ‘width variability’ ranges from highly variable (natural, unimpaired state: class I) to uniform (anthropogenically altered, completely impaired state: class V)

Each of the 25 single parameters allows for choosing one out of several parameter-states, ranging from undisturbed to totally disturbed, in comparison to predefined reference states of the respective type of river (Fig. 3). The resulting single parameters scores (classes 1–5) are averaged to obtain a score for each of the six main parameters, resulting in decimal values from 1.0 to 5.0. These six main parameter scores are aggregated (mean value) into three river sectors, i.e., river bed, riverbank, and floodplain corridor. Finally, an overall score of each stream section is calculated by aggregating the sector scores into five final classes of hydromorphological quality (I—high, II—good, III—moderate, IV—poor, V—bad) (Gellert et al. 2014; Lamberty et al. 2016). The general applicability of the LAWA-OS method to assess tropical and subtropical rivers was demonstrated by Birnbaum and Lamberty (2019).

The socio-cultural assessment follows the method developed by König (2011). This visual rapid assessment method applies five quality parameters as indicators for socio-cultural features (Kaiser 2007): visibility, reachability, accessibility, peculiarity, and amenity (Table 1 and Fig. S2). These parameters evaluate the integration of streams into the urban context and the perceptibility of its socio-cultural functions such as space for recreation or experience of nature. Each parameter is scored from 1 to 5, aggregated (mean value), and classified into an overall score of socio-cultural stream quality: I—high (1–1.8), II—good (1.81—2.6), III—moderate (2.61–3.4), IV—poor (3.41–4.2), V—bad (4.21–5).

Although the two methods only consider the aspects mentioned above and disregard other features such as diverse uses of riverine spaces or the symbolic significance of streams, it was nevertheless chosen as the method of choice. As visual rapid assessment methods, they provide reproducible results with reasonable effort.

The hydromorphological and socio-cultural quality of 60 stream sections with a total length of 15.9 km was assessed: 13 sections (section length: 500 m) at Yaque River (6.5 km), 22 sections (section length: 200 m) at Yerbabuena creek (4.4 km), and 25 sections (section length: 200 m) at Los Gatos creek (5 km) (Fig. 1). At Yaque River another 4 sections were not accessible.

### Citizen survey

To contextualize the findings of the hydromorphological and socio-cultural assessment, a citizen survey with 36 questions was conducted (Table 2 and Fig. S3) to gather basic demographic information, and to evaluate the citizens’ perception of existing BGI in Jarabacoa and their attitude towards river restoration. Together with Plan Yaque officers and volunteers, the questionnaire was adjusted to guarantee that all questions were understandable by a local audience.

**Table 2:** Parameters of the citizens survey

Citizens survey (selected parameters)	
**Household demographic information** 1. Neighborhood 2. Address 3. Age 4. Sex 5. No. of household members 6. No. of elderly members 7. No. of children 8. Time living in Jarabacoa 9. Time living in current neighborhood 10. Estimated household monthly income	**Usage of green spaces** 11. Walking time to closest green space 11.1. Where? 12. Opinion about green spaces’ quality in town 13. Opinion about green spaces’ quantity in town 14. Visit frequency to green spaces in town 15. Preferred activities when visiting green spaces^a^ 16. Preferred facilities for new public green spaces^a^
**Usage of streams as BGI** **Yaque River within urban area** 17. Walking time to closest accessible spot at Yaque River 17.1 Where?^a^ 18. Visit frequency to Yaque River within the urban area 19. Preferred activities when visiting Yaque River^a^ 20. Reasons for visiting Yaque River^a^ 21. Reasons discouraging visiting Yaque River^a^ 22. Opinion about the state of Yaque River 23. Main problems affecting Yaque River^a^	**Rivers outside the urban area** 26. Visit frequency to rivers outside the urban area 27. Time to the closest accessible river outside the urban area 27.1 Where?^a^ 28. Preferred activities when visiting rivers outside the urban area^a^ 29. Reasons for visiting rivers outside the urban area^a^ 30. Reasons discouraging visiting rivers outside the urban area^a^ 31. Opinion about the state of the rivers outside the urban area
**Yerbabuena and Los Gatos Creeks** 24. Opinion about the state of Yerbabuena and Los Gatos Creeks 25. Main problems affecting Yerbabuena and Los Gatos Creeks^a^	
**Attitudes towards restoration** 32. Previous knowledge about “river restoration” 33. Preferred interventions if river restoration were implemented^a^	34. Main benefits of restoring rivers in Jarabacoa^a^ 35. Concerns about river restoration in Jarabacoa^a^ 36. Willingness to participate in river restoration^a^

^a^In these questions, respondents could choose or provide up to three different options/answers

The survey recorded variables using ranges (e.g., daily/weekly/monthly visit frequency), predefined options (e.g., activities in green spaces), 5-point Likert scale opinions and attitudes (e.g., stream quality), and before–after scenarios illustrated by pictures (e.g., river restoration) (Iarossi 2006).

To estimate the socioeconomic status of households, one minimum Dominican wage (9400 Dominican pesos according to SIUBEN 2018), roughly equivalent to 160 euros by 2017 standards, was used as the unit to define 5 monthly average income levels: < 1 minimum wage, 1–2, 2–5, 5–10, or > 10. The gathered socio-demographic information was contrasted with the most recent census report for Jarabacoa (ONE 2012).

The sampling methodology followed the protocol of the Center for Disease Control and Prevention (CDC 2008) with adjustments due to data and logistical limitations. The universe of the survey included all inhabitants of the urban and peri-urban areas of Jarabacoa, which was 46 462 according to the most recent official figures available (Corral 2011). A sample size of 96 questionnaires yields an error margin of 0.1 and a confidence level of 95%. For a safety margin, anticipating possible errors in the survey process by the volunteer team, the sample size was increased to 160.

For a semi-stratified sampling method, a GIS map of urbanization density was generated using Google Maps (2017) satellite imagery and aerial photographs, the most recent land use plan (Corral 2011), and Plan Yaque staff’s local knowledge. With this information, the city was manually subdivided into similar high, medium, and low-density areas, checked against the density figures provided for some neighborhoods by the 2011 land use plan, and assigned 86, 65, and 25 inhabitants per hectare, respectively. The number of questionnaires per density type was defined by multiplying the total sampling number (160) by the respective density–area ratio, which acted as a weight coefficient. The survey was conducted with the local NGO, volunteers, and community leaders. For every assigned neighborhood, each surveyor would choose a random house to start, and then go to the next *n*th house (16th in compact urbanized areas and 8th in scattered-house areas), following a zig-zag pattern, until completing 10 questionnaires. After a validation process, 108 questionnaires remained.

### Prioritization of stream segments for restoration and complementary measures

The results of the stream assessment and citizen survey were applied to define and prioritize measures through a three-step process. Firstly, stream sections with good or high hydromorphological and socio-cultural potentials were identified based on their respective assessment scores. Secondly, a set of measures was selected through expert judgment by combining the aforementioned stream potentials with preferences of the population, information on land ownership, and potential costs of measures. In the third step, the proposed measures were ranked applying a set of common evaluation criteria for restoration projects, such as habitat improvement, public needs, technical feasibility, or costs (Beechie et al. 2008) (see Table 3).

**Table 3:** Prioritization of the proposed restoration and complementary measures to foster Blue-Green Infrastructure (BGI) in Jarabacoa based on their aggregated scores for each weighted prioritization criterium. Weights and scores were assigned by the authors and are shown only for demonstrative purposes to showcase the method’s general applicability

Measures		Weight	Prioritization criteria									
			Habitat improvement	Consideration of public needs	Proven technique	Upgrading of public space	Multifunctionality	Feasibility	Alignment with local development	Educational value	Low cost	Total weighted score (max. 100)
			3	3	3	3	2	2	2	1	1	
Restoration (R)	R1. Linear park Yaque–Jimenoa confluence	Score (1–5)	4	5	5	5	5	5	5	5	3	95
	R2. Riparian buffer strip Yaque River		5	5	5	5	5	3	5	5	2	93
	R3. Eco-trail Los Gatos–Jimenoa confluence		5	3	5	4	5	4	5	5	4	88
	R4. Stepping stone habitats		3	4	5	5	4	5	5	4	5	88
	R5. Small-scale constructed wetlands		5	4	5	4	3	4	5	3	3	84
	R6. Riverside park Puente Amarillo		3	4	4	4	5	4	5	4	3	80
	R7. Riverside park Yaque–Yerbabuena confluence		3	4	5	5	3	3	5	4	2	79
	R8. Private-plot revegetation Los Gatos creek		4	3	3	2	4	3	5	4	5	69
Complementary (C)	C1. Incorporation of BGI in municipal land use plan	Score (1-5)	5	5	3	5	5	3	5	3	3	86
	C2. Trails connecting green-blue spaces		1	4	5	5	3	5	5	5	3	79
	C3. Maintenance of public blue-green spaces		1	5	5	4	2	5	5	5	4	78
	C4. Wastewater pipelines and interceptors system		5	5	5	4	2	1	5	1	1	75
	C5. Artistic interventions ‘Reconnecting to our rivers’		1	4	4	5	1	4	5	5	4	71

The weighting of the prioritization criteria and the assigning of scores to these criteria for each measure are optimally carried out through multi-stakeholder dialog. Due to limited time and resources, this step was performed by the authors to showcase the method’s general applicability. Despite being highly subjective, the proposed scoring system is flexible, simple, and transparent, to allow for a participatory decision-making process.

## Results

### Hydromorphological and socio-cultural stream quality

The three assessed streams provide fundamentally different hydromorphological and socio-cultural qualities. Yaque River is the least disturbed of the three watercourses, while Los Gatos and Yerbabuena creeks exhibit high levels of anthropogenic alteration. While Yaque River is in parts a visible, accessible, and aesthetic element of Jarabacoa’s landscape, Yerbabuena and Los Gatos Creeks are largely invisible in the urban fabric, and their aesthetic quality and potential for recreation are significantly impaired.

### Yaque River

**Fig. 4:**
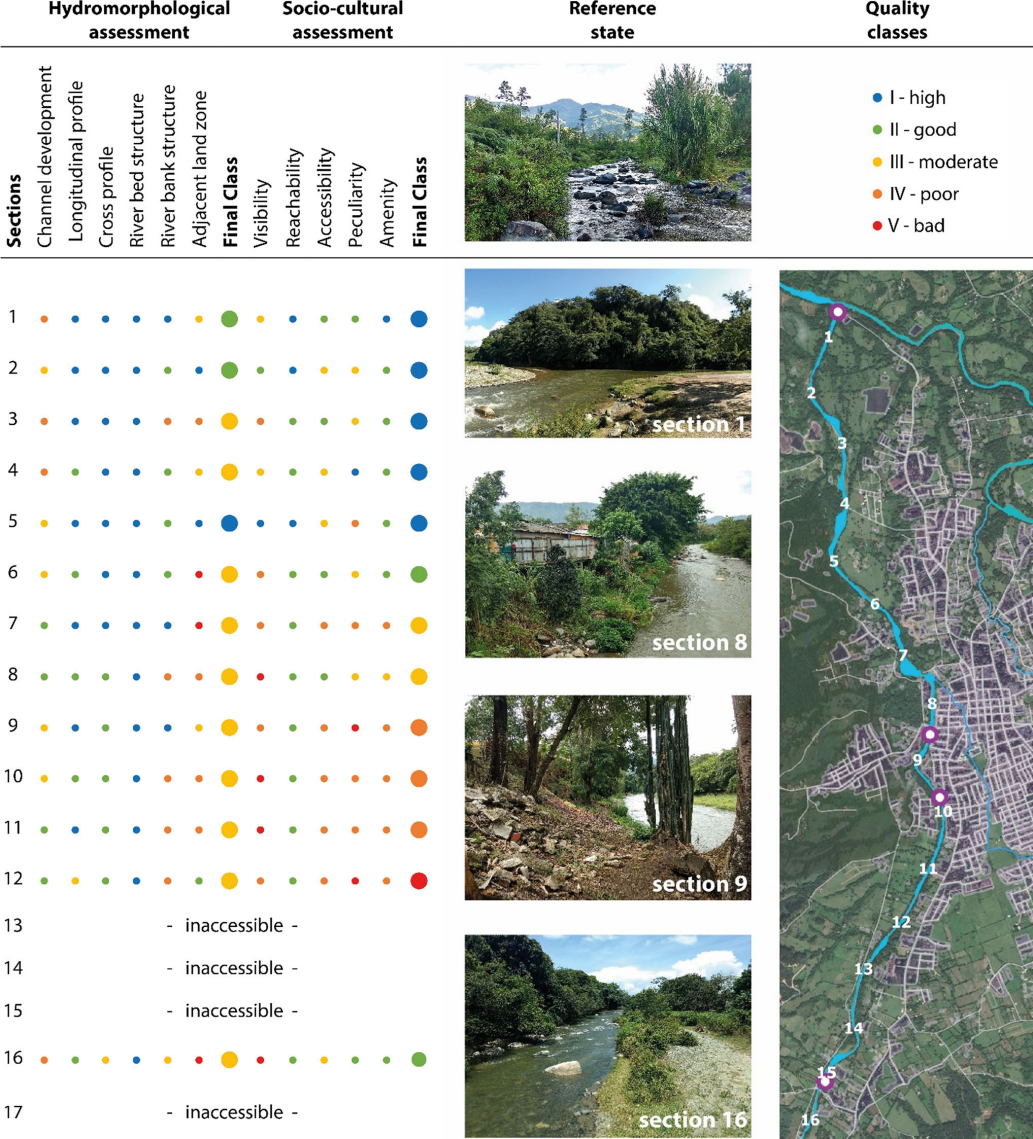
Hydromorphological (left and right river sides are aggregated) and socio-cultural assessment (only right river side is shown) results of the seventeen stream sections along the Yaque River (right). Reference state picture was taken at the Yaque River 7 km upstream from Jarabacoa (top-middle). Sections labeled as ‘inaccessible’ were not possible to assess due to large private plots impeding access to the river. The purple-white points along the Yaque River indicate the position of the exemplary photographs (middle)

In 3 of the 13 assessed sections, the Yaque River shows high to good hydromorphological quality (classes I–II) (Fig. 4). The moderate scores (class III) in the remaining 10 sections are related to changes in the floodplain corridor such as missing natural vegetation, intensive land use, and gray infrastructure in the riverine zone. The river bed itself, however, shows no to very little alteration (class I) in all 13 sections. Downstream of the city center (sections 1 to 7), the river banks on both sides are well preserved (predominately classes I–II). Higher urban density corresponds with increased anthropogenic alterations and pressures such as builtup river banks, bank protection against erosion, and infrastructure in the riverine zone.

Regarding the socio-cultural quality, 7 sections of the Yaque River score good or above (classes I–II). A near-natural scenic river spot, popular among locals and tourists (section 1), gives access to a 3-km-long walking trail towards the city center providing several semi-natural vegetated riverside spots along its course (sections 1 to 6). Lower socio-cultural scores along the trail relate to garbage and missing facilities (e.g., toilets, ramps, benches), informative boards, and maintenance. With exception of section 16 (class II), the socio-cultural quality of the remaining 6 sections is moderate to bad (classes III–V), where access is blocked by private properties and scenic values are greatly reduced by urbanization.

### Yerbabuena and Los Gatos creeks

Besides section 1 of Yerbabuena and sections 1–5 of Los Gatos (classes I–III), the loss of natural hydromorphological elements in both creeks is considerable. At Yerbabuena, 21 of the 20 assessed sections and 20 of the 25 Los Gatos sections have undergone severe hydromorphological degradation (classes IV–V) (Fig. 1). Mainly the river banks and floodplains of both creeks are severely modified due to the pressures of urbanization, such as bank protection or constructions alongside the water bodies. The river beds of the creeks show slightly better hydromorphological conditions. Although used as sewage drains, both creeks have several short (5–20 m) and a few longer (250–1000 m) segments with potential for improving hydromorphological conditions despite the existing encroachment of buildings.

Regarding their socio-cultural quality, both creeks score mainly poor to bad (classes IV–V) (Yerbabuena: 13 out of 25 sections; Los Gatos: 13 out of 22 sections), and nowhere better than moderate (class III). Despite several sections providing good reachability, visibility, and accessibility, the overall scores are reduced by very low amenity and peculiarity levels. Especially Yerbabuena creek is intensively affected by garbage, debris, feces, bad odors, and traffic noise. In large segments, this creek has been reduced to an open sewage channel. Los Gatos creek shows very similar overall scores as Yerbabuena creek, but slightly different parameter performance. As Los Gatos creek flows through private properties for most parts it is very difficult to spot and access. The sections close to the stream mouth (sections 1 to 5) score good to moderate regarding peculiarity and amenity (classes II–III). The remaining sections provide partially good to moderate visibility, accessibility, and reachability (classes II–III) but poor or bad amenity and peculiarity (classes IV–V).

### Citizens survey

The sample shows a balanced gender proportion, with a bias towards individuals above 40 years and a marked under-representation of teenagers (14–19) compared to the local census ONE (2012). Accordingly, nearly 75% of the interviewees live in households of 3 or more members, and 59% of the total sample has no children. Socioeconomically, 65% of the respondents earn a total monthly income of 2 minimum wages or less, placing them close to the poverty line (one minimum wage ≈160 euros). The vast majority of respondents (94%) are long-term local citizens (10 years or more) and thus familiar with Jarabacoa and its surroundings.

**Fig. 5:**
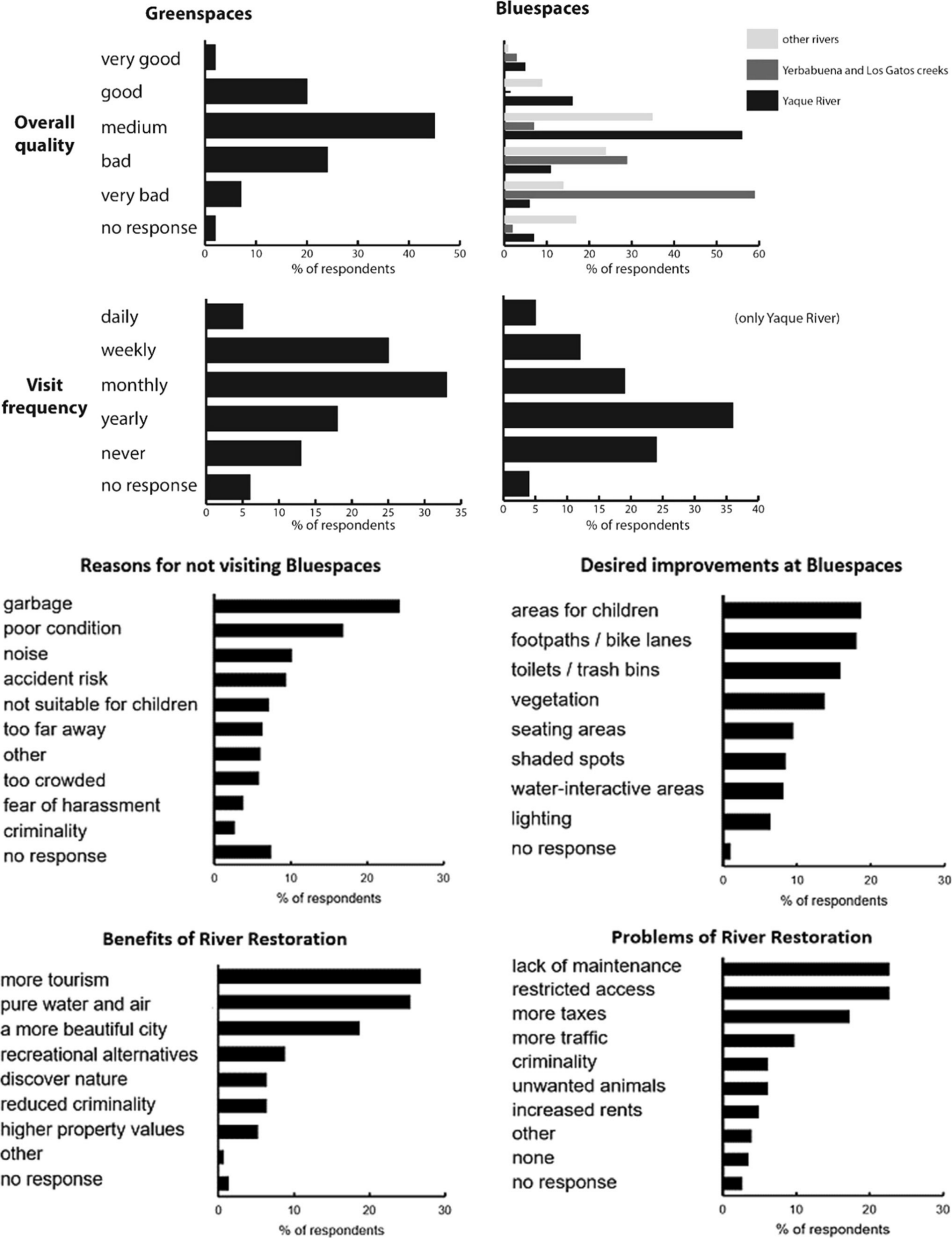
Usage and perception of green spaces and streams by Jarabacoa’s population based on 108 questionnaires of the citizens survey

Concerning green spaces, the majority of the respondents value their quantity and quality as medium or higher (Fig. 5), while perceptions about the quality of blue spaces are less favorable. Yaque River’s overall quality as blue space is considered medium or better by 48% of the respondents, and only 10% of the respondents recognize the current state of Yerbabuena and Los Gatos creeks as medium or better. The main activities in green spaces are recreational like walking, relaxing, enjoying nature, or socializing with relatives and friends.

The frequency of visits is considerably higher for green spaces than for blue spaces: 30% of the respondents visit green spaces at least once per week, while 17% do so with Yaque River and 18% with other rivers. Only 14% never visit rivers outside the urban area, while 24% never visit Yaque River within the urban area.

The respondents’ time demands to access green spaces are low: nearly 60% can access green spaces within a 10-minute walk, which is only true for 24% to reach Yaque River. Two sites along the Yaque River represent more than half of the preferred visit spots (‘Yaque–Jimenoa Confluence’ with 40% and ‘Puente Amarillo’ with 17%) which illustrates the relatively low accessibility and reachability of the river. The major reasons for not visiting Yaque River are garbage (28%), poor condition of the river (21%), and noise (12%).

The general willingness to support river restoration is high among the respondents. Some would participate in volunteer campaigns (36%), disseminate information (32%), or contribute to public discussions (20%). In contrast, options like donating money or land, ‘voting for politicians that propose restoration,’ and ‘paying a small tax’ were neglected (6% and 2%, respectively). A positive effect on tourism and improved water and air quality are considered as potential benefits of river restoration (27% and 25%, respectively), while lack of maintenance (23%), restricted access (22%), and higher taxes (17%) are seen as the main potential drawbacks.

### Restoration and complementary measures to develop blue-green infrastructure

**Fig. 6:**
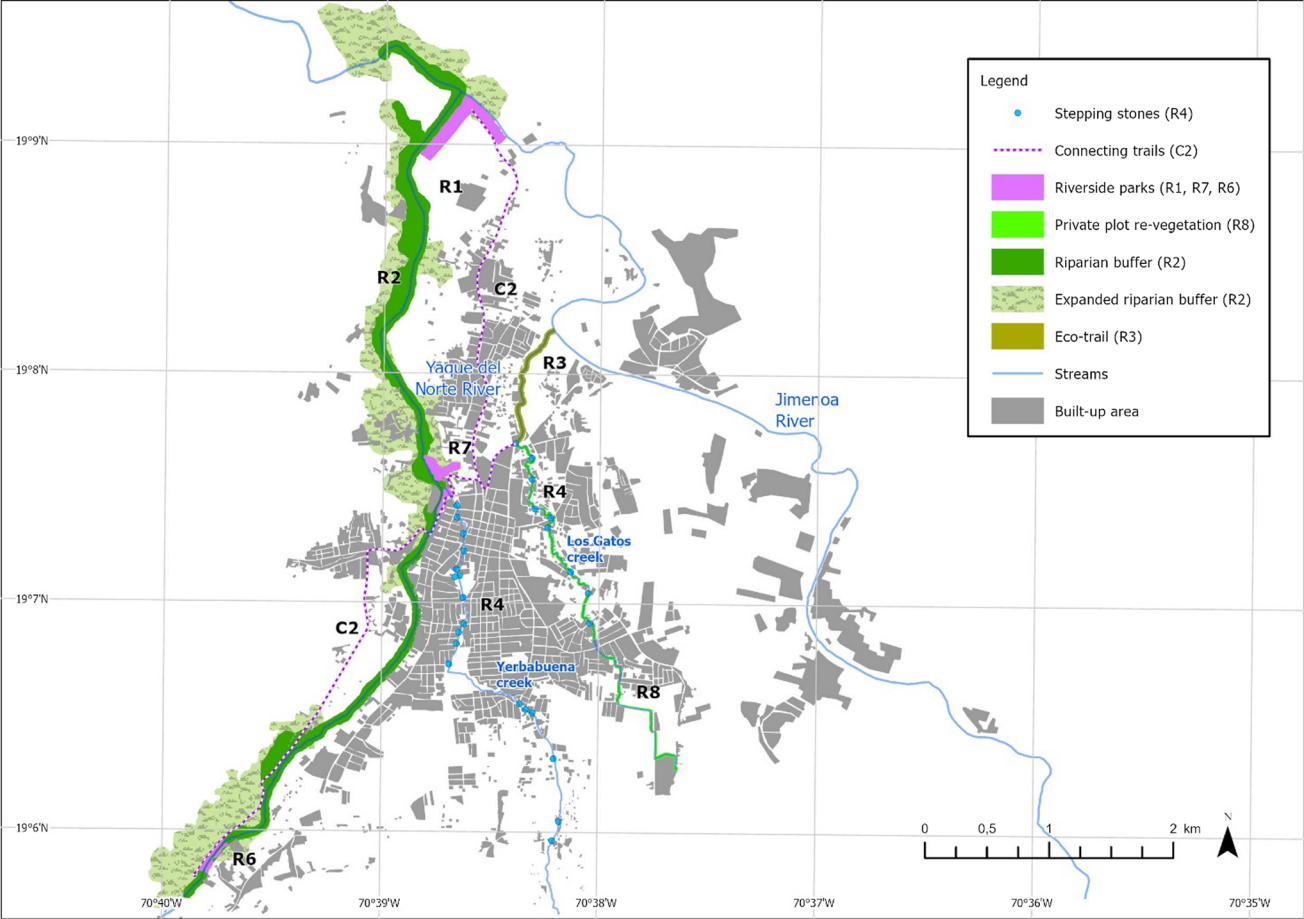
Location of the proposed restoration measures (R) and complementary measures (C) along the three assessed streams

In total, 8 restoration measures (R) and 5 complementary measures (C) are proposed to improve BGI in Jarabacoa (Table 3). The location of the restoration and complementary measures that can be spatially assigned is shown in Fig. 6. The 13 measures foster the hydromorphological and socio-cultural potential of BGI, and relate to the preferences of Jarabacoa’s population, especially the reasons for not visiting existing blue spaces and the desired improvement of such places. The numbering of the measures indicates their priority.

A linear park (R1) would connect the city center with the riverside park at the Yaque–Yerbabuena confluence (R7) incorporating elements for improved amenity and accessibility like viewpoints or water steps. A riparian buffer strip (> 30 m) should be established on both banks of the Yaque River (R2) to restore the broadleaf gallery vegetation, reduce the risk of erosion, and create additional blue-green space with direct access to the Yaque River. An additional riverside park (R6) with improved buffer strip vegetation and amenities such as seating or swimming areas would upgrade the second most visited spot at the Yaque River (Fig. 4, section 16).

At Los Gatos creek, an ecological trail to the Jimenoa–Los Gatos confluence (R3) is suggested by enforcing the existing buffer strip protection regulations. However, the main development potential of the Yerbabuena and Los Gatos creeks lies in developing small-scale ecological stepping stones (R4) and improving riverbank vegetation inside private plots (R8).

A community-based restoration program incorporated into the municipal land use plan is suggested (C1), along with linking and providing better access to all blue-green spaces by bicycle and pedestrian trails (C2), improving maintenance of blue-green spaces (C3), retrofitting wastewater infrastructure (C4), reducing direct wastewater discharge through constructed wetlands (R5), and reconnecting streams with the people and the city by designing public access spots, blue-green pocket spaces, and public gardens along the streams (C5).

## Discussion

The objective of this study is to employ a combined social–ecological rapid assessment method in a tropical low-development urban context and assess its applicability for identifying and prioritizing restoration measures. The method reveals spatially and contextually detailed information on hydromorphological and socio-cultural pressures and potentials of streams. The combination of this information with a citizen survey on perceptions of BGI allows for localization and prioritization of restoration measures.

Assessing the applicability of the method, the discussion will firstly check on the promise of bridging the gap between the ecological and social benefits potentially provided by urban BGI, and to yield applicable results. We will then assess the context of application in tropical urban settings, regarding the institutional environment and data availability. Thirdly, the claims of an effective rapid assessment are discussed, in line with the validity and consistency of results. At last, uncertainties related to the method are discussed along with some recommendations for further improvements and adaptation.

Understanding the role and importance of people–place connections, feelings of belonging, intrinsic value, and aesthetic perceptions is strategic to the resilience and transformation of social–ecological systems, as well as to restoration processes (Escalera-Reyes 2020). In that sense, it is highly advisable to complement stream assessments with a surveying or social cartography to gather key information that would otherwise be unnoticed (e.g., popular areas and activities, concerns of minorities or underrepresented groups, aesthetic preferences, risk perception). In that line, the applied method showed its potential for integrating stream assessment with societal surveying. It is understood, however, that the applied social survey lacks granularity with respect to different dimensions of social reality.

When a higher level of detail is required, a refinement of the societal assessment is advisable. Changes in perceptions, practices, and degree of place attachment along the river continuum, as well as overlapping and contradicting interests among different stakeholders, can be better captured through in-depth interviews and mental maps (Boyer et al. 2019). Similarly, a more exhaustive process to incorporate stakeholders in the scoring, weighting, and ranking of decision options can be achieved by a multi-criteria analysis as designed by Ruangpan et al. (2020) or Geodesign tools such as interactive scenario maps and real-time impact assessment (Gottwald et al. 2020).

In general, the citizen survey results largely coincide with the visual assessment of streams, but aesthetics and water quality were more relevant to citizens than structural or ecological aspects (e.g., loss of ecological integrity). More natural blue-green spaces tend to be perceived as aesthetically better, but as Junker and Buchecker (2008) have pointed out, citizen’s lay perceptions may greatly differ from experts’ ecological or environmental standards. Moreover, citizens relate in complex ways to and deem important also informal greenspaces such as street verges, water sites, lots, railways, and brownfields (Rupprecht and Byrne 2014).

In line with this, the public perception of BGI in Jarabacoa was unexpectedly good, and the quality and quantity of existing blue-green spaces were considered neither bad nor insufficient. Designated green spaces in Jarabacoa account for less than 900 m^2^ and consist mainly of small parks and squares with little or no green. Often, respondents referred to neighborhoods or streets with few trees or vacant plots as the closest green space to their homes, regardless of whether they are inaccessible or unsuitable for public use. Additionally, the relatively small size of the builtup area (around 10 km^2^) allowing citizens to reach rural areas within a very short distance or time, and the regular use of non-designated greenspaces along the Yaque del Norte and Jimenoa rivers might also contribute to explaining the perceived amount and quality of greenspaces in town.

The method has proved to be applicable in the context of a small tropical town. It provides spatially explicit information about the hydromorphological and socio-cultural status of each stream and its segments and differences in the type and extent of alterations and allows to differentiate well-preserved, moderately altered, and critically degraded areas regarding both components, thus delivering a consistent basis for prioritizing restoration measures. Such results achieved in a low-data environment are promising, and the application towards prioritization of interventions has proven feasible, despite a lack of citizens and other stakeholders’ participation in that step. Field surveying, especially rapid visual assessment methods, emerge as valid in Global South countries given the fact that neither expert knowledge nor costly equipment is required (Beißler and Hack 2019). This is particularly relevant in the case of local governments, NGOs, and groups of citizens willing to engage in participatory restoration processes since they have proven to be crucial pieces of the stakeholder constellation to NbS design and implementation (Zingraff-Hamed et al. 2020).

Regarding the claim of a rapid assessment and the efficiency of the method, the fieldwork could be completed in less than four weeks by one expert mapping the streams, with the support of a local NGO mainly for the survey. Insofar, the assessment yields fast and comprehensive data, if generalized in parts as a trade-off of any rapid assessment approach. The low human resources demand in numbers and necessary expertise allows for a first comprehensive survey even under limited funds and expertise.

Nevertheless, there are several uncertainties inherent to visual assessment methods that must be considered (Belletti et al. 2015): selection of sufficient and representative reference sites can be problematic, and choosing natural sites is prone to errors due to unknown distant disturbances or past events; high-status conditions tend to be associated with maximum morphological diversity, although homogeneous river morphology occurs naturally as well; regional biases may occur when applying these methods under diverging natural pre-conditions.

Given such uncertainties, and from the experiences mentioned in the results, we recommend to adjust the method to improve its transferability. Regarding the parameter setup and the scoring process, the method should be further refined. Some considerations should be taken when applying it in other locations. Here, we highlight the importance of identifying the natural or near-natural state of streams under investigation considering the historic land use of the region before conducting the assessment. Accordingly, we recommend adjusting the scorecard, if necessary, to address the expected local river type characteristics and to allow regional comparison of assessment results. In that sense, tailoring the method to local conditions and incorporating remotely sensed data and GIS analysis are appropriate possibilities to further adapt the method to the tropical context (Belletti et al. 2015; Beißler and Hack 2019).

We also suggest improving the resolution of the socio-cultural parameters to better characterize the aspects that might foster or limit citizens’ interaction with the river and its integration with the urban fabric, and the inclusion of several new parameters to better address some of the most widespread problems in Global South cities, such as poor urban planning, deficient facilities maintenance, and insecurity.

The revised parameters should aim at balancing the scoring process to reduce the weight of contradictory qualities. For example, a well-preserved gallery forest hampers the direct view of the water, thus yielding a lower score. Similarly, reduced accessibility is desirable in certain areas where citizens should be discouraged or prevented from direct access for safety reasons (e.g., flash flood risk) or to reduce wildlife disturbance (e.g., nesting/feeding areas for birds). Insofar, the scorecard mirrors classical conflicts of interests, and we suggest modifying these parameters so that they evaluate the extent to which the river is visible and accessible only in segments where no superior goals contradict. Also, depending on the river size, citizens can enjoy them by having an open view of the floodplain (not merely the river itself), as well as being able to walk, bike, etc., over or along the floodplain, without accessing it. Therefore, a more detailed assessment of social river connectivity (*sensu* Kondolf and Pinto 2017) is advisable.

Regarding the scoring process, it is important to keep the two scores for socio-cultural and hydromorphological quality independent, to keep transparent potentially contradicting ecological and urban qualities. For large and medium-sized rivers, aggregation of both riversides is not advisable in the case of socio-cultural parameters, as heterogeneous land ownership and arbitrary planning decisions may create major differences between one bank and the other.

Going further to assess the role of the method in the wider institutional context, several social and cultural factors can both foster or hamper the current restoration efforts. Restoration, especially in an urban context, implies not only achieving ecological and hydromorphological transformations, but—very importantly—changes in mindsets, institutional arrangements, and policies, without which it is impossible to plan, design, reach agreements, and implement restoration. Protection areas along rivers and streams are already mandatory in the Dominican Republic. According to the National Environmental Law and the Municipal Land Use Plan, 30 and 50 m strips, respectively, must not be urbanized (Corral 2011). These regulations, however, are not systematically enforced. Institutional weakness also manifests itself in the lack of efficiency and continuity in public administration, the poor state of infrastructure and services provision, lack of long-term planning, and abandonment of unfinished projects.

In the survey, perceived problems affecting rivers were connected to governmental responsibilities such as ‘lack of interest by the authorities’ and ‘lack of maintenance.’ In regard to restoration experiences in other Latin American countries, da Cruz e Sousa and Ríos-Touma (2017) indicate that changes in political agenda often result in a complete reorientation of the administration, impeding successful long-term efforts and negatively impacting community trust. While dynamics of distrust and resistance to change are often seen as the sole responsibility of local authorities, in reality, all stakeholders share responsibilities in shaping the urban system. Its proper functioning highly depends on effective communication between stakeholders and their awareness of the impacts of their actions (Abarca-Guerrero et al. 2013). For instance, the presence of garbage was consistently mentioned as a problem and reason not to visit rivers, while respondents alluded to ‘lack of citizen awareness’ as most relevant in affecting the Yaque River and the two streams. Not surprisingly, during fieldwork, citizens could repeatedly be observed throwing garbage, construction debris, and other objects directly into the rivers without facing any social sanction.

From its very beginnings, restoration originated in citizens’ support and leadership desiring a different relationship between nature and society. Thus, the level and quality of civil society participation and involvement in planning processes become key aspects in restoration success (Buijs 2009; Wohl et al. 2015). Relevant progress has been made in Jarabacoa in recent years. One major step forward was the explicit interest in integrated basin management actions, constituted in the Plan for the Development of the Yaque River basin (Plan Yaque 2020).

Even under very different governance approaches, the assessment and planning process can effectively work as an interface where discussions, opinions, and solution negotiations across different disciplines and among different stakeholders constitute the basis for successful NbS implementation (Wang et al., 2021). Plan Yaque, organized as a multi-stakeholder NGO integrated by more than 30 governmental institutions and civil society organizations, serves as a prototype for multi-stakeholder river management in the Dominican Republic. Since 2013, it has already conducted several participatory workshops, bringing together practitioners, public officials, community leaders, and volunteer citizens to discuss river restoration under a community-based problem-solving approach. It has successfully installed decentralized wastewater plants (i.e., constructed wetlands) in several neighborhoods in a joint effort with neighbor committees that have donated the plots needed and participated in the design and construction. In that sense, Plan Yaque enjoys multilateral and public trust as a well-established stakeholder platform (which, by the way, facilitated the survey).

Despite the behavior mentioned above, the survey results clearly showed citizens are backing and willing to support and actively contribute to restoration activities. Certainly, Plan Yaque’s work has influenced people’s perceptions, and during the river visual assessment, random citizens approached the team to talk, offered their help, or made suggestions about the rivers’ situation. Also, environmental awareness campaigns conducted by other institutions and organizations, increasing media coverage, and growing public debates on this topic may contribute as well to citizens’ awareness. It has been shown that the stronger the feelings of attachment and belonging of local communities to the ecosystems and territory they live in, the higher the chances of success of changing a situation considered irreversible and initiating new possible pathways (Escalera-Reyes 2020). In that sense, the study identified public trust and interest in Plan Yaque’s actions as one of the biggest potentials. Plan Yaque has successfully managed to achieve community involvement, long-term planning, and multi-stakeholder engagement beyond governmental institutions’ abilities. If communities become an active party in the restoration process, Jarabacoa can achieve sustainable restoration and enhancement of its rivers and BGI.

## Conclusion

The proposed rapid assessment method has demonstrated to be efficient, offering potentials for a comprehensive assessment of rivers and streams across entire metropolitan areas. Despite having been developed for a different geographical and social context, the method proved to be applicable in the Global South providing spatially explicit information on hydromorphological characteristics and socio-cultural conditions of urban streams.

Necessary improvement of the method relates to the review of indicators for socio-cultural conditions of streams, to ensure a broader view of the societal services provided by rivers and creeks. Furthermore, the hydromorphological assessment must be linked to a reference system for rivers that is valid for the tropics to be able to upscale the method and transfer it to other locations.

The method is designed to identify feasible restoration measures to promote NbS. In its current state, however, the method lacks explicit participatory elements to involve stakeholders in the planning process.

## Supplementary Information

Below is the link to the electronic supplementary material.

Supplementary material 1 (PDF 2002 KB)

Supplementary material 2 (XLSX 54 KB)

Supplementary material 3 (XLSX 17 KB)
