# Supplementary material for: Hydromorphological and socio-cultural assessment of urban rivers to promote nature-based solutions in Jarabacoa, Dominican Republic
Source: Ambio. 2021 Jun 1;50(8):1414–30. doi: 10.1007/s13280-021-01565-3 (PMC8249590; doi:10.1007/s13280-021-01565-3)
Supplement: Supplementary file 1 — Supplementary material 1 (PDF 2002 KB) [file 13280_2021_1565_MOESM1_ESM.pdf]

Ambio

Electronic Supplementary Material
This supplementary material has not been peer reviewed.

Title: **Hydromorphological and socio-cultural assessment of urban rivers to promote nature-based solutions in Jarabacoa, Dominican Republic**

Authors: Gonzalo Pradilla, Georg Lamberty, Johannes Hamhaber

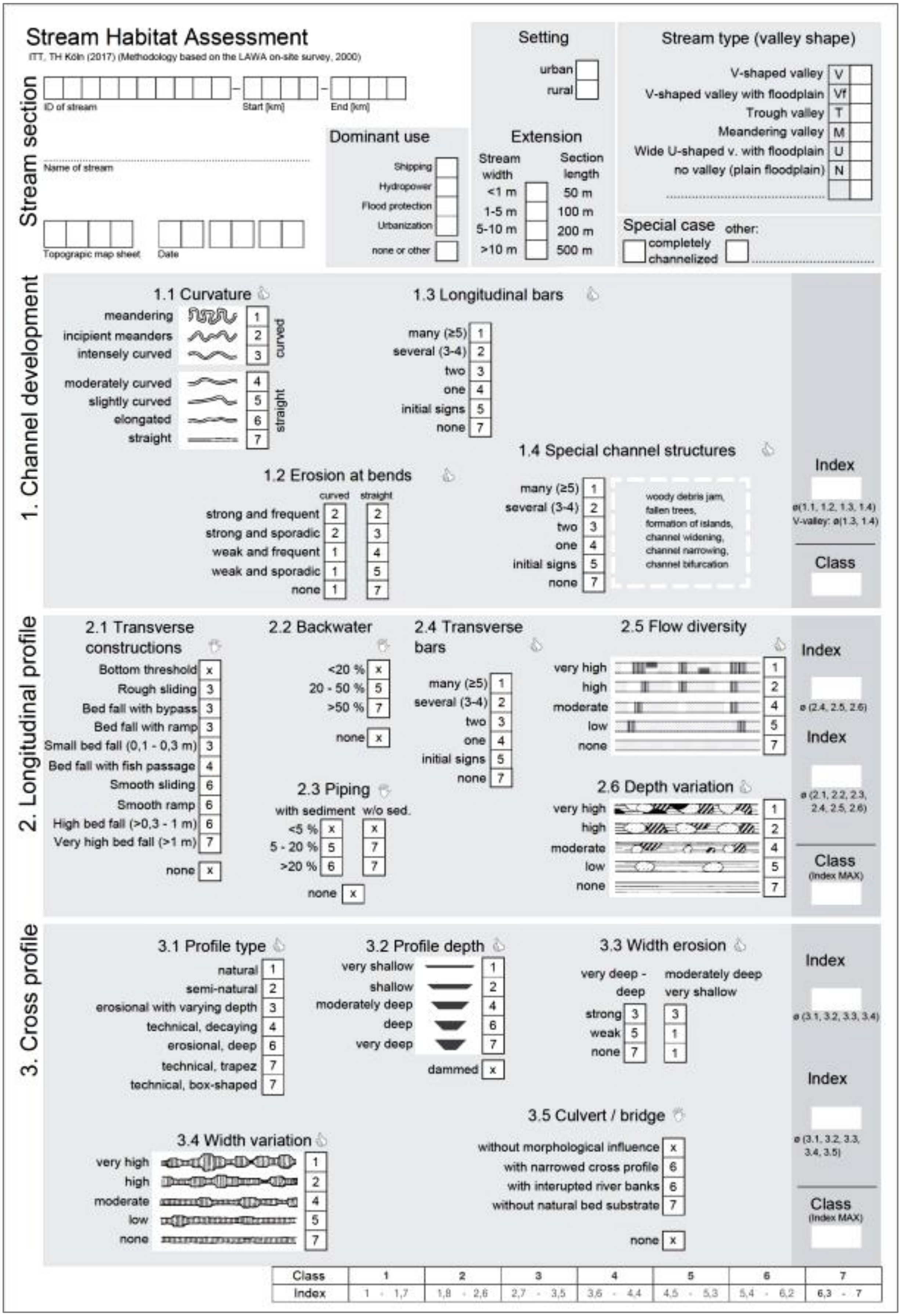

Stream Habitat Assessment

ITT, TH Köln (2017) (Methodology based on the LAWA on-site survey, 2000)

Stream section

ID of stream – Start [km] – End [km]

Name of stream

Topograpic map sheet | Date

Dominant use

- Shipping
- Hydropower
- Flood protection
- Urbanization
- none or other

Setting

- urban
- rural

Extension

Stream width		Section length
<1 m		50 m
1-5 m		100 m
5-10 m		200 m
>10 m		500 m

Stream type (valley shape)

V-shaped valley	V
V-shaped valley with floodplain	Vf
Trough valley	T
Meandering valley	M
Wide U-shaped v. with floodplain	U
no valley (plain floodplain)	N
....	

Special case

completely channelized

other: ....

1. Channel development

1.1 Curvature

meandering	1
incipient meanders	2
intensely curved	3
moderately curved	4
slightly curved	5
elongated	6
straight	7

(1–3: curved; 4–7: straight)

1.2 Erosion at bends

	curved	straight
strong and frequent	2	2
strong and sporadic	2	3
weak and frequent	1	4
weak and sporadic	1	5
none	1	7

1.3 Longitudinal bars

many (≥5)	1
several (3-4)	2
two	3
one	4
initial signs	5
none	7

1.4 Special channel structures

many (≥5)	1
several (3-4)	2
two	3
one	4
initial signs	5
none	7

woody debris jam, fallen trees, formation of islands, channel widening, channel narrowing, channel bifurcation

Index: ø(1.1, 1.2, 1.3, 1.4); V-valley: ø(1.3, 1.4)

Class

2. Longitudinal profile

2.1 Transverse constructions

Bottom threshold	x
Rough sliding	3
Bed fall with bypass	3
Bed fall with ramp	3
Small bed fall (0,1 - 0,3 m)	3
Bed fall with fish passage	4
Smooth sliding	6
Smooth ramp	6
High bed fall (>0,3 - 1 m)	6
Very high bed fall (>1 m)	7
none	x

2.2 Backwater

<20 %	x
20 - 50 %	5
>50 %	7
none	x

2.3 Piping

	with sediment	w/o sed.
<5 %	x	x
5 - 20 %	5	7
>20 %	6	7
none	x	

2.4 Transverse bars

many (≥5)	1
several (3-4)	2
two	3
one	4
initial signs	5
none	7

2.5 Flow diversity

very high	1
high	2
moderate	4
low	5
none	7

2.6 Depth variation

very high	1
high	2
moderate	4
low	5
none	7

Index: ø (2.4, 2.5, 2.6)

Index: ø (2.1, 2.2, 2.3, 2.4, 2.5, 2.6)

Class (Index MAX)

3. Cross profile

3.1 Profile type

natural	1
semi-natural	2
erosional with varying depth	3
technical, decaying	4
erosional, deep	6
technical, trapez	7
technical, box-shaped	7

3.2 Profile depth

very shallow	1
shallow	2
moderately deep	4
deep	6
very deep	7
dammed	x

3.3 Width erosion

	very deep - deep	moderately deep very shallow
strong	3	3
weak	5	1
none	7	1

3.4 Width variation

very high	1
high	2
moderate	4
low	5
none	7

3.5 Culvert / bridge

without morphological influence	x
with narrowed cross profile	6
with interupted river banks	6
without natural bed substrate	7
none	x

Index: ø (3.1, 3.2, 3.3, 3.4)

Index: ø (3.1, 3.2, 3.3, 3.4, 3.5)

Class (Index MAX)

Class	1	2	3	4	5	6	7
Index	1 - 1,7	1,8 - 2,6	2,7 - 3,5	3,6 - 4,4	4,5 - 5,3	5,4 - 6,2	6,3 - 7

Figure S1. Stream Hydromorphological and Habitat Assessment form applied to conduct the visual on-site survey (figure continues below).

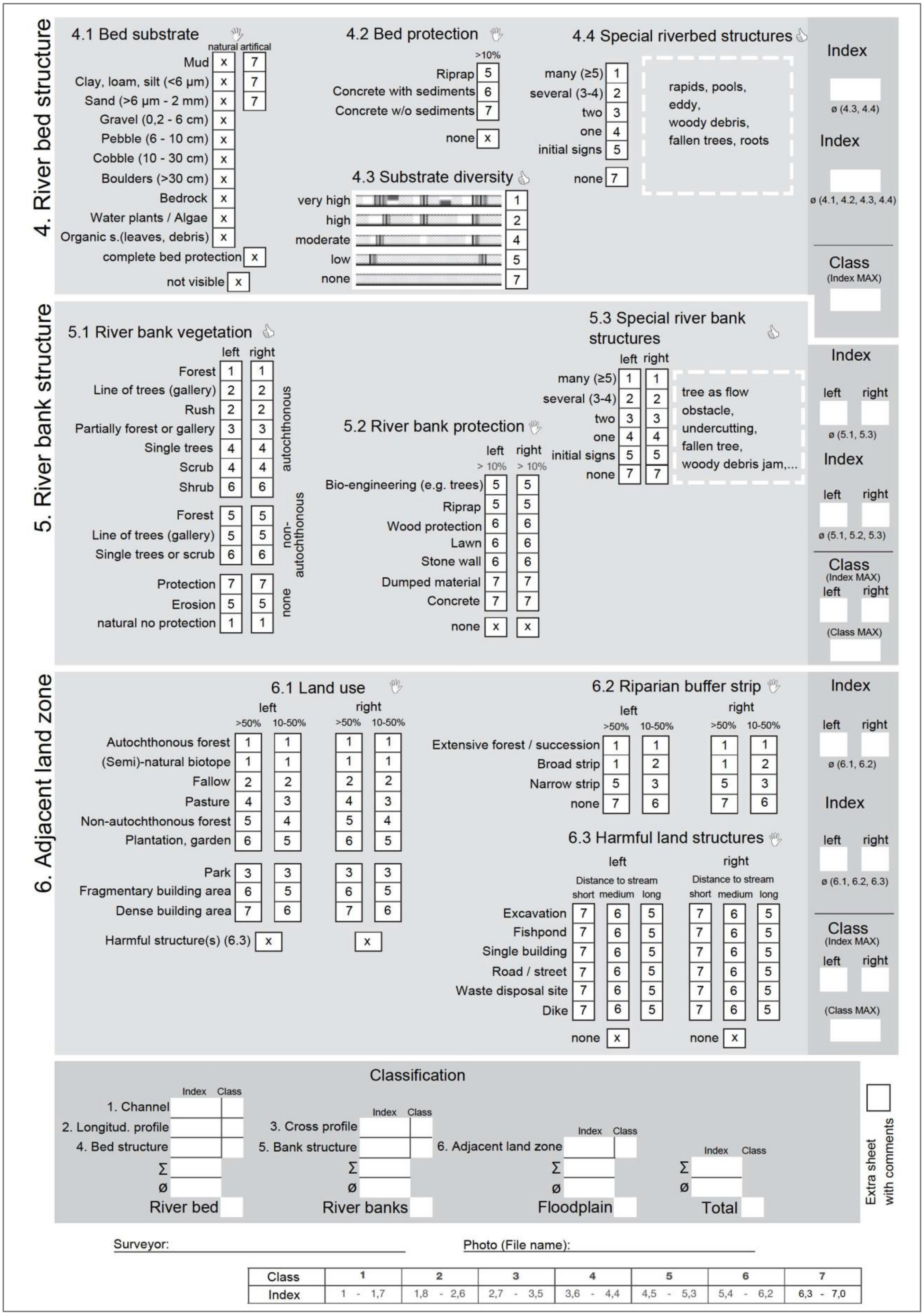

4. River bed structure

4.1 Bed substrate

	natural	artifical
Mud	x	7
Clay, loam, silt (<6 µm)	x	7
Sand (>6 µm - 2 mm)	x	7
Gravel (0,2 - 6 cm)	x	
Pebble (6 - 10 cm)	x	
Cobble (10 - 30 cm)	x	
Boulders (>30 cm)	x	
Bedrock	x	
Water plants / Algae	x	
Organic s.(leaves, debris)	x	
complete bed protection	x	
not visible	x	

4.2 Bed protection

	>10%
Riprap	5
Concrete with sediments	6
Concrete w/o sediments	7
none	x

4.3 Substrate diversity

very high	1
high	2
moderate	4
low	5
none	7

4.4 Special riverbed structures

many (≥5)	1
several (3-4)	2
two	3
one	4
initial signs	5
none	7

rapids, pools, eddy, woody debris, fallen trees, roots

Index ø (4.3, 4.4)

Index ø (4.1, 4.2, 4.3, 4.4)

Class (Index MAX)

5. River bank structure

5.1 River bank vegetation

	left	right	
Forest	1	1	autochthonous
Line of trees (gallery)	2	2	autochthonous
Rush	2	2	autochthonous
Partially forest or gallery	3	3	autochthonous
Single trees	4	4	autochthonous
Scrub	4	4	autochthonous
Shrub	6	6	autochthonous
Forest	5	5	non-autochthonous
Line of trees (gallery)	5	5	non-autochthonous
Single trees or scrub	6	6	non-autochthonous
Protection	7	7	none
Erosion	5	5	none
natural no protection	1	1	none

5.2 River bank protection

	left > 10%	right > 10%
Bio-engineering (e.g. trees)	5	5
Riprap	5	5
Wood protection	6	6
Lawn	6	6
Stone wall	6	6
Dumped material	7	7
Concrete	7	7
none	x	x

5.3 Special river bank structures

	left	right
many (≥5)	1	1
several (3-4)	2	2
two	3	3
one	4	4
initial signs	5	5
none	7	7

tree as flow obstacle, undercutting, fallen tree, woody debris jam,...

Index left right ø (5.1, 5.3)

Index left right ø (5.1, 5.2, 5.3)

Class (Index MAX) left right (Class MAX)

6. Adjacent land zone

6.1 Land use

	left >50%	left 10-50%	right >50%	right 10-50%
Autochthonous forest	1	1	1	1
(Semi)-natural biotope	1	1	1	1
Fallow	2	2	2	2
Pasture	4	3	4	3
Non-autochthonous forest	5	4	5	4
Plantation, garden	6	5	6	5
Park	3	3	3	3
Fragmentary building area	6	5	6	5
Dense building area	7	6	7	6
Harmful structure(s) (6.3)	x		x	

6.2 Riparian buffer strip

	left >50%	left 10-50%	right >50%	right 10-50%
Extensive forest / succession	1	1	1	1
Broad strip	1	2	1	2
Narrow strip	5	3	5	3
none	7	6	7	6

6.3 Harmful land structures

	left short	left medium	left long	right short	right medium	right long
	Distance to stream			Distance to stream		
Excavation	7	6	5	7	6	5
Fishpond	7	6	5	7	6	5
Single building	7	6	5	7	6	5
Road / street	7	6	5	7	6	5
Waste disposal site	7	6	5	7	6	5
Dike	7	6	5	7	6	5
none	x			x		

Index left right ø (6.1, 6.2)

Index left right ø (6.1, 6.2, 6.3)

Class (Index MAX) left right (Class MAX)

Classification

	Index	Class
1. Channel		
2. Longitud. profile		
4. Bed structure		
Σ		
ø		
River bed		

	Index	Class
3. Cross profile		
5. Bank structure		
Σ		
ø		
River banks		

	Index	Class
6. Adjacent land zone		
Σ		
ø		
Floodplain		

	Index	Class
Σ		
ø		
Total		

Extra sheet with comments

Surveyor:

Photo (File name):

Class	1	2	3	4	5	6	7
Index	1 - 1,7	1,8 - 2,6	2,7 - 3,5	3,6 - 4,4	4,5 - 5,3	5,4 - 6,2	6,3 - 7,0

Figure S1. Stream Hydromorphological and Habitat Assessment form applied to conduct the visual on-site survey.

SOCIO-CULTURAL URBAN STREAM ASSESSMENT			
**VISIBILITY**		**Left**	**Right**
Very good	> double water width	☐	☐
Good	= double water width or < but multiple viewpoints	☐	☐
Moderate	< double water width and few viewing points	☐	☐
Bad	walls, buildings, dense vegetation or similar hamper the view	☐	☐
Not visible	river flows underground	☐	☐
**REACHABILITY**			
Very good	continuous pedestrian/bike path + public transport access +/or parking area	☐	☐
Good	partial pedestrian/bike path + public transport access +/or parking area	☐	☐
Moderate	pedestrian/bike paths lead only punctually to the river, or there are pedestrian and/or cycle paths but no public transport or parking	☐	☐
Bad	only unsuitable paths or roads for pedestrians/bicycles lead to the waterway	☐	☐
Not reachable	no paths or streets by or leading to the river	☐	☐
**ACCESSIBILITY**			
Direct	direct contact with the water is possible without significant obstacles. The access may e.g. be relieved by shallow banks or stairs.	☐	☐
Moderate	accessible with reasonable effort; no obstacles or if obstacles multiple contact points	☐	☐
Limited	possible but difficult (vegetation, steep bank); or water is inaccessible (fences, bushes, or walls), but there are punctual access points	☐	☐
Very limited	no access options or effort/risk is too high (river behind buildings, fences, walls too high)	☐	☐
Not accessible	river flows underground	☐	☐
**PECULIARITY***			
Very high	river has a special recognition value / special elements create a very attractive and unique ambience	☐	☐
High	high quality and recognition value / elements + structures create an attractive environment as a whole	☐	☐
Medium	some attractive elements + structures and an overall impression/aesthetic	☐	☐
Poor	seldom characteristic elements/structures, no uniform overall impression	☐	☐
Very low	there are no characteristic elements and the area is unattractive	☐	☐
**AMENITY**			
Very high	many positive factors and the disturbing ones only play a very minor role	☐	☐
High	predominantly positive factors are present, but single disturbing factors occur	☐	☐
Medium	positive and disturbing factors balance each other	☐	☐
Bad	some positive factors but negative ones outweigh them	☐	☐
Very low	Being at the river is unpleasant or not possible at all due to many negative factors	☐	☐

Figure S2. Socio-Cultural Stream Assessment form applied to conduct the visual on-site survey (figure continues below).

Guiding criteria and additional information

Positive		
Contemplation spaces	▪ Peace and seclusion	☐
	▪ Natural diversity	☐
	▪ View of the running water is possible	☐
Gathering spaces	▪ Attractive shores with an impressive scenery	☐
	▪ Seating places (e.g. benches, cafes) by the water	☐
	▪ Historical elements (e.g. mills, bridges, weirs, etc.)	☐
	▪	
	▪ Artworks thematically or spatially related to the water	☐
	▪ Nature trails or informative boards	☐
Experiential spaces	▪ Flow-calmed shallow water areas, suitable for splashing and playing	☐
	▪ Bathing places with a water depth of> 1m with good water quality	☐
	▪ Playing and lying areas (do not have to be officially designated)	☐
	▪ Barbecue areas (only officially designated, nowild bonfires)	☐
	▪ Regularly emptied trash containers	☐
	▪ Toilets	☐
	▪ Sport facilities	☐
	▪ Rental of boats, bicycles, horses etc.	☐

Negative	
▪ Noise (e.g. traffic)	☐
▪ Odor (sewage, industry, road)	☐
▪ Mosquitoes	☐
▪ Animal excrement	☐
▪ Garbage shore / in the water	☐
▪ Wild vegetation	☐
▪ Insecurity	☐
▪ Bad water quality	☐

**Use**	Current:	
	Historic (original):	
**Aesthetic conservation status**	Very good	☐
	Good	☐
	Rudimentary	☐
**Functional conservation status**	Fully functional	☐
	Partially functional	☐
	Functionless, but readable	☐
	Functionless	☐
**Experientiality**	Accessibility	☐
	Function visible and observable (e.g. mill wheel)	☐
	Cultural-historical value highlighted (board, sign, etc.)	☐

***Peculiarity aspects**

o historical buildings and transversal structures
o materials and architectural styles
o architectural features
o backdrop effect of the environment
o visual relations
o course and structures of the water body
o faunistic and floristic peculiarities
o usages of the river
o space and dimensions
o artistic figures and sculptures
o sensual stimulation through light, colors, smell and sounds

NOTES:

____

____

____

Figure S2. Socio-Cultural Stream Assessment form applied to conduct the visual on-site survey.

Thank you for agreeing to take part in this survey to better understand the perceptions of the inhabitants of Jarabacoa on the green spaces and rivers in the city. The following survey is conducted under the cooperation of the Cologne University of Applied Sciences (Germany) and Plan Yaque (Dominican Republic). It will take you 15 to 20 minutes to complete the questionnaire. Be assured that all the answers and information you provide will be kept in the strictest confidentiality and your personal information will not be disclosed.

Questionnaire No. **Date:** / /18 **D.K.** Don't know **N.R.** No response

1. Neighborhood: **2. Address:**

**3. Age**	14-19 ☐	20-29 ☐	30-39 ☐	40-49 ☐	50-59 ☐	> 60 ☐	N.R. ☐	D.K. ☐
**4. Sex**	Masculine ☐	Feminine ☐	Other ☐	N.R. ☐	D.K. ☐			

5. No. of household members: ____ **6. No. of elderly members (> 65 years old):** ____ **7. No. of children (< 14 years old):** ____

**8. How long have you been living in Jarabacoa? (years)**	0-1 ☐	1-3 ☐	3-10 ☐	10-20 ☐	20-30 ☐	> 30 ☐	N.R. ☐	D.K. ☐
**9. How long have you been living in your current neighborhood? (years)**	0-1 ☐	1-3 ☐	3-10 ☐	10-20 ☐	20-30 ☐	> 30 ☐	N.R. ☐	D.K. ☐

**10. What is the approximate total monthly income of your household? (DOP)**	< $9.400 ☐	$9.400 - 18.800 ☐	$18.800 - 47.000 ☐	$47.000 - 94.000 ☐	> $94.000 ☐	N.R. ☐	D.K. ☐

GREEN SPACES: open air spaces with abundant vegetation and/or trees

**11. What is the walking time from your home to the closest green space inside the city?** *(please indicate it on the map)*	Less than 5 min ☐	5 to 10 min ☐	10 to 20 min ☐	20 to 30 min ☐	More than 30 min ☐	D.K. ☐	N.R. ☐
**12. How do you rate the overall quality of green spaces inside the city?**	Very good ☐	Good ☐	Medium ☐	Bad ☐	Very bad ☐	D.K. ☐	N.R. ☐
**13. How do you rate the overall quantity of green spaces inside the city?**	Very good ☐	Good ☐	Medium ☐	Bad ☐	Very bad ☐	D.K. ☐	N.R. ☐
**14. How often do you visit green spaces inside the city?**	Almost every day ☐	Few times a week ☐	Few times a month ☐	Few times a year ☐	Never ☐	D.K. ☐	N.R. ☐

**15. What are your preferred activities when visiting green spaces?** *(max. 3)*	Walk / relax ☐	Enjoy Nature ☐	Time with family/friend ☐	Practice sports ☐	Eat/drink something ☐	Play with my children ☐	Educational activities ☐	Other: ☐		D.K. ☐	N.R. ☐
**16. If the city was to create a new public green space, which of the following would you prefer to have there?** *(max. 3)*	Music / Disco-theque ☐	Natural areas ☐	Sports faci-lities ☐	Food and drinking ☐	Areas for children ☐	Cultural / Artistic ☐	Interactive installations ☐	Bike lanes ☐	Other: ☐	D.K. ☐	N.R. ☐

YAQUE RIVER WITHIN THE URBAN AREA (between “La Confluencia” and “Puente Amarillo”)

**17. What is the <u>walking</u> time from your home to the closest accessible spot at Yaque River?** *(please indicate on the map)*	Less than 5 min ☐	5 to 10 ☐	10 to 20 ☐	20 to 30 ☐	> 30 min ☐					D.K. ☐	N.R. ☐
**18. How often do visit the river in this area?**	Almost every day ☐	Couple times a week ☐	Couple times monthly ☐	Couple times yearly ☐	Never (go to 22) ☐					D.K. ☐	N.R. ☐
**19. What are your preferred activities when visiting the river in this area?** *(Max. 3)*	Walk / relax ☐	Enjoy Nature ☐	Fishing ☐	Practice sports ☐	Eat drink ☐	Educa-tional activities ☐	Swim ☐	Time with family/ friends ☐	Other: ☐	D.K. ☐	N.R. ☐
**20. What motivates you to visit the river in this area?** *(max. 3)*	Close to home ☐	Natural environ ment ☐	Clean-liness ☐	Low cost ☐	Quietn ess ☐	My children can play ☐	No other options available ☐	Good facilities ☐	Lack of options in town ☐	Other: ☐	D.K. ☐	N.R. ☐
**21. What discourages you from visiting the river in this area?** *(Max. 3)*	Too distant ☐	Poor state of river ☐	Garbage ☐	Noise ☐	Risk of harass-ment ☐	Inadequa te for children ☐	Crime ☐	Risk of accidents ☐	Too crowded ☐	Other: ☐	D.K. ☐	N.R. ☐
**22. How do you rate the overall state of the river in this area?**	Very good ☐	Good ☐	Medium ☐	Bad ☐	Very bad ☐					D.K. ☐	N.R. ☐
**23. In your opinion, what are the most important problems affecting Yaque River in this area?** *(Max. 3)*	Water pollution ☐	Lack of interested by local institutions ☐	Garbage ☐	Lack of vegetation ☐	Lack of main-tenance ☐	Lack of citizens’ awareness ☐	Alteration of the river’s structure ☐	Other ☐		D.K. ☐	N.R. ☐

YERBABUENA AND LOS GATOS CREEKS

**24. How do you rate the overall state of Yerbabuena and Los Gatos Creeks?**	Very Good ☐	Good ☐	Medium ☐	Bad ☐	Very bad ☐				D.K. ☐	N.R. ☐
**25. In your opinion, what are the most important problems affecting these creeks?** *(Max. 3)*	Water pollution ☐	Lack of interested by local institutions ☐	Garbage ☐	Lack of vegetation ☐	Lack of main-tenance ☐	Lack of citizens' awareness ☐	Alteration of the river's structure ☐	Other ☐	D.K. ☐	N.R. ☐

RIVERS OUTSIDE THE URBAN AREA (including Yaque River rural sections)

**26. How often do you visit rivers outside the urban area?**	Almost every day ☐	Few times a week ☐	Few times a month ☐	Few times a year ☐	Never (go to #23) ☐					D.K. ☐	N.R. ☐
**27. How long does it take you go from home to the closest accessible river outside the urban area?** *(please indicate on the map)*	5 to 10 min walk ☐	10 to 20 min walk ☐	10 to 20 min bus / car ☐	20 to 30 min bus /car ☐	> 30 min bus /car ☐					D.K. ☐	N.R. ☐
**28. What are your preferred activities when visiting rivers outside the urban area?** *(Max. 3)*	Walk / relax ☐	Enjoy Nature ☐	Fishing ☐	Practice sports ☐	Eat /drink ☐	Time with family/ friends ☐	Educa-tional activities ☐	Swim ☐	Other: ☐	D.K. ☐	N.R. ☐
**29. What motivates you to visit any of these rivers?** *(Max. 3)*	Close to home ☐	Natural environ ment ☐	Clean-liness ☐	Low cost ☐	Quiet-ness ☐	My children can play ☐	No other options available ☐	Good facilities ☐	Lack of options in town ☐	Other: ☐	D.K. ☐	N.R. ☐
**30. What discourages you from visiting any of these rivers?** *(Max. 3)*	Too distant ☐	Bad environ -mental quality ☐	Dirty / Garbage ☐	No-where to sit ☐	Noise ☐	No facilities (WC, food) ☐	Crime ☐	Risky ☐	Other: ☐	D.K. ☐	N.R. ☐
**31. How do you rate the overall state of the rivers outside the urban area?**	Very Good ☐	Good ☐	Medium ☐	Bad ☐	Very bad ☐						

**32. Do you know the concept "river restoration"?**	I understand it very well ☐ *(please explain below)*	I have a basic idea ☐ *(please explain below)*	Heard of it but don't know what it is ☐	Never heard of it ☐
	(In a few words) I understand that river restoration is: ____ ____			

Before continuing with the questionnaire, show the sample images to the interviewee.

Figure S3. Citizen survey questionnaire on blue-green infrastructure use and perception.

**33. If river restoration were planned in Jarabacoa, which interventions would you consider most important?** *(Max. 3)*	Walking/ bicycling paths ☐	Lighting ☐	Areas for children ☐	Water interactive facilities ☐	Benches ☐	Shade ☐	Toilettes, trash bins ☐	Vegeta-tion ☐	Other: ☐	D.K. ☐	N.R. ☐
**34. In your opinion, what would be the main benefits of restoring the rivers in the city?** *(Max. 3)*	More tourism ☐	Lower crime ☐	Discover Nature ☐	Higher property values ☐	More recreation al activities ☐	Clearer water and cleaner air ☐	A more beautiful city ☐	Other: ☐		D.K. ☐	N.R. ☐
**35. Would any of the following be a concern?** *(Max. 3)*	Restricted public access ☐	Higher taxes ☐	Traffic and parking problems ☐	Lack of maintenance ☐	Crimina-lity Insecurity ☐	Un-wanted animals / pests ☐	Increased rent prices ☐	Other: ☐		D.K. ☐	N.R. ☐
**36. Would you be willing to participate in the restoration of the river and its green spaces?** *(choose 2 indicating 1st and 2nd)*	Volunteer work (e.g. tree planting, cleaning) ☐	Promote and share infor-mation ☐	Contribute to public discussion s ☐	Support politicians proposing restoration ☐	Paying a small tax ☐	Donation in cash or land ☐	Other: ☐	Volunteer work (e.g. tree planting, cleaning) ☐		D.K. ☐	N.R. ☐

Thank you for your participation! In case that our team finds your case particularly representative or interesting, would you be willing to be contacted again for an interview? If so, please provide us with a phone number and/or email: ____.
